# Pruritus and Fatigue in Patients With Metabolic Dysfunction-Associated Steatotic Liver Disease: A Study of Turkish Patients From the Global NASH/MASH Registry

**DOI:** 10.14309/ctg.0000000000000844

**Published:** 2025-04-28

**Authors:** Yusuf Yilmaz, Caglayan Keklikkiran, Andrei Racila, Maria Stepanova, Zobair M. Younossi

**Affiliations:** 1 The Global NASH Council, Center for Outcomes Research in Liver Diseases, Wahington, District of Columbia, USA;; 2 Institute of Gastroenterology, Marmara University, İstanbul, Türkiye;; 3 Department of Gastroenterology, School of Medicine, Recep Tayyip Erdoğan University, Rize, Türkiye;; 4 Beatty Liver and Obesity Research Program, Inova Health System, Falls Church, Virginia, USA;; 5 Center for Outcomes Research in Liver Disease, Washington, District of Columbia, USA.

**Keywords:** metabolic dysfunction-associated steatotic liver disease, pruritus, fatigue, patient-reported outcomes, Global Liver Registry

## Abstract

**INTRODUCTION:**

Despite being underappreciated, pruritus and fatigue are not uncommon in metabolic dysfunction-associated steatotic liver disease (MASLD). In this prospective registry-based study, we sought to evaluate the prevalence, predictors, and impact of these symptoms on patient-reported outcomes (PROs) in patients with MASLD from Türkiye, a country with one of the highest burdens of MASLD globally.

**METHODS:**

A total of 1,874 Turkish patients from the Global Liver Registry were included. Significant pruritus and fatigue were defined using the Chronic Liver Disease Questionnaire-nonalcoholic steatohepatitis and the Functional Assessment of Chronic Illness Therapy-Fatigue, respectively. PROs were assessed using the Chronic Liver Disease Questionnaire-nonalcoholic steatohepatitis, Functional Assessment of Chronic Illness Therapy-Fatigue, and the Work Productivity and Activity Impairment: Specific Health Problem questionnaire.

**RESULTS:**

The prevalence of significant pruritus and fatigue was 37% and 33%, respectively. Both symptoms were significantly associated with female sex, type 2 diabetes, depression, abdominal pain, and lack of regular exercise. Patients with these symptoms had significantly worse PROs, with impairments up to 31% (all *P* < 0.0001). Over 1–2 years, symptoms persisted in 47%–52% of patients with baseline pruritus and 33%−39% with baseline fatigue. Independent predictors of a higher likelihood of resolution of pruritus included the absence of fatigue at baseline and the lack of abdominal pain, with odds ratios of 0.36 (95% confidence interval: 0.22−0.59) and 0.60 (95% confidence interval: 0.39−0.91), respectively (both *P* < 0.05).

**DISCUSSION:**

Pruritus and fatigue are common in MASLD, significantly impairing PROs and often persisting over time. Regular assessment and management are essential to improve patients' well-being and functioning.

## Graphical abstract

**Figure:**
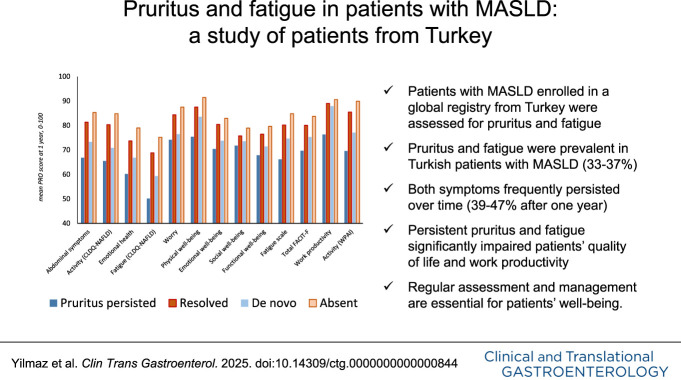

## INTRODUCTION

The global prevalence of metabolic dysfunction-associated steatotic liver disease (MASLD) is rising at an alarming rate, mirroring the worldwide increase in obesity and type 2 diabetes (1). Currently affecting 38% of adults globally (1), MASLD has become the most common chronic liver disease, with projections indicating it may affect more than 55% of adults by 2040 (2). Contrary to earlier beliefs that MASLD was largely asymptomatic, recent research has revealed its significant impact on health-related quality of life (HRQoL) (3,4) and patient-reported outcomes (5,6). These measures have become crucial endpoints in both clinical trials and real-world studies, reflecting a growing emphasis on patient-centered care in hepatology (7).

Among the various symptoms associated with MASLD, pruritus and fatigue have gained recognition as 2 of the most prevalent and burdensome (8). These symptoms exert a profound influence on PROs, significantly affecting patients' HRQoL and daily functioning. A study of 1,669 patients with advanced steatohepatitis from the safety and efficacy of selonsertib in adults with nonalcoholic steatohepatitis (NASH) and bridging fibrosis phase 3 clinical trial revealed that 27% reported pruritus, while 33% experienced clinically significant fatigue (8). Pruritus was linked to female sex, lower serum albumin levels, and depression, whereas fatigue was independently associated with type 2 diabetes, depression, and nervous system disorders (8). Both pruritus and fatigue were found to negatively affect PROs across all measured domains, with fatigue demonstrating a more pronounced effect (8). Notably, fatigue in MASLD has also been associated with increased mortality risk (9) and may significantly impede efforts to implement crucial lifestyle modifications, particularly increased physical activity, which are essential for effective MASLD management (10). Regarding pruritus, a multicenter study by Oeda et al (11) reported a prevalence of 44.7% in a MASLD population, while Boehlig et al (12) found that one-fifth of MASLD patients experienced moderate to severe itching. The latter study also identified significant associations between moderate-to-severe pruritus and diabetes mellitus, depression, and anxiety (12).

Despite increasing awareness of the clinical and patient-centered burden posed by pruritus and fatigue in MASLD, these issues are still not fully recognized, and their effects on PROs remain insufficiently researched. To address this knowledge gap, we designed this study to assess the prevalence, predictors, and PRO correlates of significant pruritus and fatigue using data from the Global Nonalcoholic Steatohepatitis/MASH Registry (GNR) (13,14). Our focus was specifically on Turkish data because Türkiye has one of the highest burdens of MASLD worldwide, making it a significant public health priority (15).

## PATIENTS AND METHODS

### Study population

The GNR was established in 2014 as a collaborative initiative among researchers from more than 20 countries involved in the Global Nonalcoholic Steatohepatitis Council (13,14). Investigators interested in setting up a GNR site within their respective countries obtained approval from their local Institutional Review Board or equivalent supervisory body to enroll and consent patients. According to the GNR protocol (13,14), eligible participants included patients with a confirmed diagnosis of chronic liver disease (CLD) from a predefined list of etiologies, specifically chronic hepatitis B, chronic hepatitis C, and MASLD or nonalcoholic steatohepatitis (NASH), who were seen at participating clinical sites. Patients younger than 18 years were excluded, as were those with either etiologies of CLD not on the predefined list or with multiple etiologies. In addition, pregnant women, patients with a history of decompensated liver disease, hepatocellular carcinoma, and previous liver transplantation were not eligible for enrollment. Finally, those unwilling or unable to provide informed consent were also excluded from participation. For this study, we selected Global Liver Registry TM (GLR) enrollees with MASLD or NASH specifically from Türkiye. This focused approach allows for a detailed examination of these conditions within a population known to have a high prevalence of MASLD (15). The study received approval from the Western Institutional Review Board and from the respective Institutional Review Boards or equivalent supervisory institutions at each participating site. All research was conducted in accordance with both the Declarations of Helsinki and Istanbul, and all patients provided informed consent before enrollment in the GLR.

### Data collection

The data collection process encompassed comprehensive patient information, including demographic characteristics (age, sex, ethnicity, and enrollment site), recent anthropometric measurements (height and weight), pertinent medical history, lifestyle factors, liver imaging results, histopathological findings (biopsy results), and recent laboratory parameters (hepatic enzyme levels and platelet count). Most of the participants also completed validated PRO questionnaires, assessing HRQoL and various aspects of daily functioning (6–8,13,14). Annual follow-up evaluations were recommended for all study participants, with PRO scores collected at each subsequent visit. Trained personnel at each study site completed standardized clinical data collection forms, and all data were securely stored in a centralized repository on the sponsor's server.

### Definitions

In this study, obesity was defined according to World Health Organization criteria, with participants classified as obese if their body mass index (BMI) exceeded 30 kg/m^2^ and lean if their BMI was below 25 kg/m^2^. To assess hepatic fibrosis, we used multiple criteria to enhance accuracy. The Fibrosis-4 index, a validated noninvasive test, was calculated using age, alanine aminotransferase, aspartate aminotransferase, and platelet count (16). Participants were classified as having advanced fibrosis if they met any of the following criteria (17): Fibrosis-4 score ≥2.67, liver stiffness ≥12 kPa as measured by transient elastography (when available), or a documented history of cirrhosis based on clinical, histological, or radiological evidence.

### Patient-reported outcomes

We used 3 widely used and extensively validated PRO instruments: the Chronic Liver Disease Questionnaire-NASH (CLDQ-NASH) (18), the Functional Assessment of Chronic Illness Therapy-Fatigue (FACIT-F) (19), and the Work Productivity and Activity Impairment: Specific Health Problem (WPAI:SHP) questionnaire (20). Participants self-administered these instruments in their native language using validated translations, ensuring linguistic and cultural equivalence. The CLDQ-NASH, a disease-specific instrument, comprises 36 items across 6 key domains: abdominal symptoms, fatigue, systemic symptoms, activity, emotional function, and worry. Responses to each question are scored on a 7-point Likert scale, with higher scores representing better health. Domain scores are calculated by averaging the respective item scores (18). The FACIT-F is a comprehensive instrument designed to assess fatigue and its impact on patients with chronic illnesses. It comprises 4 core domains and a dedicated Fatigue Scale. The total FACIT-F score, ranging from 0 to 160, is calculated by summing all domain scores, with higher scores indicating better health status (19). The WPAI:SHP is a versatile tool that evaluates the impact of health issues on work productivity and daily activities. For employed patients, it measures both absenteeism (work hours missed due to health problems) and presenteeism (self-reported reduced productivity while at work). The questionnaire also assesses impairment in nonwork activities for all patients. Unlike other PRO instruments in this study, WPAI scores range from 0 to 1, with higher scores reflecting greater impairment in work productivity and activity (20). To facilitate interpretation and comparison, the PRO scores were linearly transformed from their original scales to a standardized 0–100 scale, with higher scores consistently indicating better health status (21).

### Definition of significant pruritus and fatigue

Pruritus was assessed using the CLDQ-NASH instrument, which includes the item: “How much of the time have you been troubled by itching during the last 2 weeks?” Responses range from 1 (“All of the time”) to 7 (“None of the time”) on a 7-point Likert scale (8). For this study, significant pruritus was defined as a score of 4 or lower, corresponding to experiencing itching at least “Some of the time.” Significant fatigue was defined as a FACIT-F score of 30 or below (22).

### Statistical analysis

All collected demographic and clinical parameters, as well as PRO scores, were summarized using appropriate descriptive statistics. Continuous variables were presented as mean ± SD, whereas categorical variables were expressed as frequency (percentage). Comparisons between groups were conducted using the Wilcoxon rank-sum test for continuous variables and the Pearson χ^2^ test for categorical variables. To identify independent predictors of significant pruritus and fatigue, as well as the resolution of these symptoms in individuals who had them at baseline and the development of de novo pruritus or fatigue, multivariable logistic regression analyses were performed. All statistical analyses were performed using SAS, version 9.4 (SAS Institute, Cary, NC). A 2-sided *P* value < 0.05 was considered statistically significant.

The study received approval from the Western Institutional Review Board and from the respective Institutional Review Boards or equivalent supervisory institutions at each participating site. All patients provided informed consent before enrollment in the GLR.

## RESULTS

### Patient characteristics

The study included a total of 1,874 patients with MASLD (Table 1). The prevalence of significant pruritus was 37%, whereas significant fatigue was reported by 33% of the participants. The study sample had a mean age of 49 years, with a SD of 12 years. Sex distribution showed that 49% of the participants were male. Employment status indicated that 40% of the participants were used. Notably, 72% of the participants were classified as obese, while 4% were categorized as lean. Regarding comorbidities, 49% of the participants had type 2 diabetes. Advanced fibrosis was observed in 19% of the study sample. Mental health assessments revealed that 43% of the participants reported a history of anxiety, and 18% had depression. Furthermore, 54% of the participants had clinically overt fatigue documented in their medical history. Finally, 40% of the participants reported engaging in regular exercise, defined as at least 30 minutes of physical activity at least 3 times per week.

**Table 1.** Demographic and clinical characteristics of MASLD patients stratified by the presence of significant pruritus

	Significant pruritus	No significant pruritus	*P*	All MASLD
No. of patients	684 (36.5%)	1,190 (63.5%)		1,874
Age, yr	49.5 ± 11.4	48.7 ± 11.6	0.10	49.0 ± 11.5
Male sex	275 (40.2%)	643 (54.0%)	<0.0001	918 (49.0%)
Employed	246 (36.0%)	507 (42.6%)	0.0046	753 (40.2%)
BMI, kg/m^2^	34.8 ± 6.5	33.7 ± 6.2	0.0001	34.1 ± 6.3
Obese (BMI > 30 kg/m^2^)	518 (75.8%)	825 (69.6%)	0.0036	1,343 (71.9%)
Lean (BMI < 25 kg/m^2^)	23 (3.4%)	47 (4.0%)	0.51	70 (3.7%)
Type 2 diabetes	380 (55.6%)	539 (45.3%)	<0.0001	919 (49.1%)
FIB-4 score	1.21 ± 1.03	1.24 ± 1.18	0.51	1.23 ± 1.12
Liver stiffness, kPa	10.1 ± 9.5	9.20 ± 7.52	0.18	9.53 ± 8.30
Advanced fibrosis^a^	141 (20.6%)	208 (17.5%)	0.10	349 (18.6%)
Past medical history				
Anxiety or panic disorders	346 (50.6%)	458 (38.5%)	<0.0001	804 (42.9%)
Depression	170 (24.9%)	162 (13.6%)	<0.0001	332 (17.7%)
Clinically overt fatigue^b^	454 (66.5%)	554 (46.6%)	<0.0001	1,008 (53.9%)
Abdominal pain	234 (34.2%)	211 (17.7%)	<0.0001	445 (23.8%)
Cancer (any)	41 (6.0%)	60 (5.0%)	0.38	101 (5.4%)
Hypertension	304 (44.4%)	458 (38.6%)	0.0125	762 (40.7%)
Hyperlipidemia	298 (43.7%)	435 (36.6%)	0.0027	733 (39.2%)
Sleep apnea	139 (21.0%)	175 (15.0%)	0.0011	314 (17.2%)
Moderate alcohol use	53 (7.7%)	95 (8.0%)	0.85	148 (7.9%)
Regular exercise (≥30 min ≥3/wk)	237 (34.8%)	511 (43.0%)	0.0005	748 (40.0%)
Current smoking	133 (19.4%)	217 (18.3%)	0.52	350 (18.7%)
Significant pruritus, fatigue				
Significant pruritus at 1-yr	206 (47.1%)	149 (18.3%)	<0.0001	355 (28.3%)
Significant pruritus at 2-yr	165 (52.1%)	136 (22.0%)	<0.0001	301 (32.2%)
Significant fatigue at baseline	340 (49.8%)	283 (23.8%)	<0.0001	623 (33.3%)
Significant fatigue at 1-yr	112 (25.6%)	95 (11.6%)	<0.0001	207 (16.5%)
Significant fatigue at 2-yr	61 (19.2%)	66 (10.7%)	0.0003	127 (13.6%)

Continuous data are expressed as means ± SD, whereas categorical data are presented as frequency counts (percentages).

BMI, body mass index; FIB-4, Fibrosis-4; MASLD, metabolic dysfunction-associated steatotic liver disease.

a Defined as FIB-4 ≥2.67 or liver stiffness ≥12 kPa.

b Defined as fatigue or asthenia mentioned in medical history.

### Correlates of significant pruritus

In comparison with MASLD patients without significant pruritus, those with significant pruritus were more likely to be female, less likely to be employed, and had higher BMI. In addition, they had a higher prevalence of type 2 diabetes, anxiety, depression, clinically overt fatigue, abdominal pain, hypertension, hyperlipidemia, and sleep apnea (all *P* < 0.01). Notably, these differences were observed despite similar age profiles between the 2 groups, as detailed in Table 1. Furthermore, MASLD patients with significant pruritus reported less regular exercise (35% vs 43%, *P* = 0.0005) and demonstrated significantly impaired PROs as assessed by the CLDQ-NASH, the FACIT-F, and the WPAI:SHP instruments (all but one *P* < 0.0001). The most pronounced impairments were observed in nonpruritus domains of the CLDQ-NASH, including Abdominal symptoms, Activity/energy, Emotional health, and Fatigue, with reductions ranging from −15% to −16% of the PRO range size. Similarly, significant impairments were noted in the Fatigue Scale of FACIT-F (−16%) and work productivity (−16%), as outlined in Table 2. A strong association was also observed between significant pruritus and significant fatigue, with 50% of patients with significant pruritus also experiencing significant fatigue, compared with 24% of those without significant pruritus (*P* < 0.0001; Table 1).

**Table 2.** Patient-reported outcomes of MASLD patients stratified by the presence of significant pruritus

Score (range)	Significant pruritus	No significant pruritus	*P*	All MASLD
CLDQ-NASH				
Abdominal symptoms (1–7)	4.36 ± 1.58	5.30 ± 1.48	<0.0001	4.95 ± 1.58
Activity/energy (1–7)	4.47 ± 1.39	5.40 ± 1.26	<0.0001	5.06 ± 1.38
Emotional health (1–7)	4.13 ± 1.36	5.05 ± 1.26	<0.0001	4.72 ± 1.37
Fatigue (1–7)	3.59 ± 1.45	4.56 ± 1.42	<0.0001	4.21 ± 1.51
Systemic symptoms (1–7)	3.69 ± 1.23	5.17 ± 1.15	<0.0001	4.63 ± 1.38
Worry (1–7)	4.75 ± 1.51	5.51 ± 1.35	<0.0001	5.24 ± 1.46
Total (1–7)	4.17 ± 1.13	5.16 ± 1.04	<0.0001	4.80 ± 1.17
FACIT-F				
Physical well-being (0–28)	19.0 ± 6.2	22.8 ± 5.1	<0.0001	21.4 ± 5.8
Emotional well-being (0–24)	14.3 ± 5.2	16.7 ± 4.9	<0.0001	15.9 ± 5.1
Social well-being (0–28)	19.4 ± 6.1	20.6 ± 6.1	<0.0001	20.2 ± 6.1
Functional well-being (0–28)	17.9 ± 5.7	20.5 ± 5.4	<0.0001	19.6 ± 5.7
Fatigue scale (0–52)	29.2 ± 12.7	37.4 ± 11.3	<0.0001	34.4 ± 12.5
Total (0–160)	99.8 ± 27.9	118.0 ± 24.5	<0.0001	111.4 ± 27.2
WPAI:SHP				
Work productivity impairment (1-0)	0.302 ± 0.336	0.141 ± 0.248	<0.0001	0.194 ± 0.290
Absenteeism (1-0)	0.038 ± 0.140	0.028 ± 0.119	0.29	0.031 ± 0.126
Presenteeism (1-0)	0.267 ± 0.306	0.114 ± 0.219	<0.0001	0.164 ± 0.261
Activity impairment (1-0)	0.300 ± 0.314	0.158 ± 0.256	<0.0001	0.210 ± 0.286

Data are expressed as means ± SD, whereas categorical data are presented as frequency counts (percentages).

CLDQ-NASH, Chronic Liver Disease Questionnaire-NASH; FACIT-F, Functional Assessment of Chronic Illness Therapy-Fatigue; MASLD, metabolic dysfunction-associated steatotic liver disease; WPAI:SHP, Work Productivity and Activity Impairment, Specific Health Problem.

### Longitudinal findings on significant pruritus

**Figure 1.**
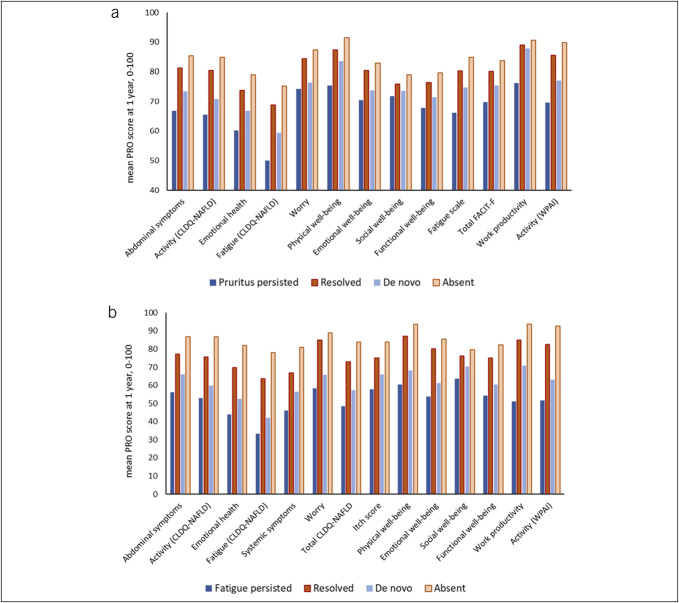
Comparison of mean patient-reported outcome scores in MASLD patients experiencing (**a**) pruritus and (**b**) fatigue at baseline vs 1-year follow-up. CLDQ-NASH, chronic liver disease Questionnaire-NASH; FACIT-F, functional assessment of chronic illness therapy-fatigue; MASLD, metabolic dysfunction-associated steatotic liver disease; PRO, patient-reported outcome; WPAI, work productivity and activity impairment, specific health problem.

Among the MASLD patients with a 1-year follow-up (n = 1,253, representing 67% of the baseline sample), a higher prevalence of significant pruritus was observed in those who had significant pruritus at baseline. Specifically, 47% of patients with significant pruritus at baseline continued to experience significant pruritus at the 1-year follow-up, compared with 18% of patients who developed de novo significant pruritus despite not having the condition at baseline (*P* < 0.0001; Table 1). For the subsample with a 2-year follow-up (n = 935, representing 50% of the baseline sample), the rates of persistent vs de novo pruritus were similar to those observed at the 1-year mark. Notably, 52% of patients with significant pruritus at baseline continued to experience significant pruritus after 2 years, while 22% developed de novo significant pruritus (*P* < 0.0001; Table 1). Across 4 patient groups defined by the presence of significant pruritus at baseline and 1-year follow-up, the PRO scores varied significantly. These subsets included patients who never experienced pruritus (53%), those who developed de novo pruritus (12%), those whose baseline pruritus resolved (18%), and those with persistent pruritus at both time points (16%). Notably, the PRO scores were the lowest among patients with persistent pruritus, followed by those who developed de novo pruritus (*P* < 0.0001; Figure 1a). Conversely, patients who never experienced significant pruritus had higher PRO scores at the 1-year follow-up compared with those of patients whose baseline pruritus resolved. Specifically, impairments of up to −6% were noted in 4 nonpruritus domains of the CLDQ-NASH, 3 domains of the FACIT-F, and the Activity domain of the WPAI (*P* < 0.05; Figure 1a).

### Correlates of significant fatigue

A significant association was observed between pruritus and fatigue in the study cohort. Specifically, 48% of the sample exhibited neither condition, 18% experienced significant pruritus without significant fatigue, 15% had significant fatigue without significant pruritus, and 18% suffered from both conditions. The odds ratio (OR) for the association was 3.2 (95% confidence interval [CI]: 2.6−3.9, *P* < 0.0001). Similar to the demographic characteristics of patients with significant pruritus, those with significant fatigue were more likely to be female, less likely to be employed, had higher BMI, and more frequently experienced type 2 diabetes and other nonhepatic comorbidities. In addition, these patients engaged in less regular exercise compared with those without significant fatigue, with all these differences being statistically significant (*P* < 0.01; Table 3). Patients experiencing significant fatigue also reported substantial impairments in PRO scores. The most pronounced impairments were observed across all nonfatigue domains of CLDQ-NASH, with reductions of up to 28% of the score range. Similarly, significant declines were noted in all nonfatigue domains of FACIT-F, reaching up to 31%, and in work productivity and activity as measured by the WPAI questionnaire, with reductions of up to 29% (Table 4).

**Table 3.** Demographic and clinical characteristics of MASLD patients stratified by the presence of significant fatigue

	Significant fatigue	No significant fatigue	*P*
No. of patients	623 (33.3%)	1,249 (66.7%)	
Age, yr	48.0 ± 11.4	49.5 ± 11.6	0.0089
Male sex	216 (34.7%)	702 (56.2%)	<0.0001
Employed	194 (31.2%)	558 (44.7%)	<0.0001
BMI, kg/m^2^	35.2 ± 6.8	33.4 ± 6.0	<0.0001
Obese (BMI > 30 kg/m^2^)	482 (77.5%)	858 (68.9%)	0.0001
Lean (BMI < 25 kg/m^2^)	21 (3.4%)	49 (3.9%)	0.55
Type 2 diabetes	341 (54.7%)	578 (46.3%)	0.0006
FIB-4 score	1.17 ± 1.03	1.26 ± 1.17	0.0219
Liver stiffness, kPa	9.81 ± 9.30	9.38 ± 7.77	0.67
Advanced fibrosis^a^	123 (19.8%)	226 (18.1%)	0.38
Past medical history			
Anxiety or panic disorders	396 (63.6%)	407 (32.6%)	<0.0001
Depression	191 (30.7%)	141 (11.3%)	<0.0001
Clinically overt fatigue^b^	538 (86.4%)	468 (37.6%)	<0.0001
Abdominal pain	249 (40.0%)	194 (15.5%)	<0.0001
Cancer (any)	46 (7.4%)	55 (4.4%)	0.0071
Hypertension	275 (44.1%)	486 (39.0%)	0.0320
Hyperlipidemia	279 (44.9%)	453 (36.4%)	0.0004
Sleep apnea	155 (25.6%)	158 (12.9%)	<0.0001
Moderate alcohol use	41 (6.6%)	107 (8.6%)	0.13
Regular exercise (≥30 min ≥3/wk)	202 (32.6%)	545 (43.7%)	<0.0001
Current smoking	136 (21.8%)	215 (17.2%)	0.0163
Significant pruritus, fatigue			
Significant pruritus at baseline	340 (54.6%)	343 (27.5%)	<0.0001
Significant pruritus at 1-yr	171 (41.9%)	184 (21.8%)	<0.0001
Significant pruritus at 2-yr	129 (43.4%)	172 (27.0%)	<0.0001
Significant fatigue at 1-yr	157 (38.5%)	50 (5.9%)	<0.0001
Significant fatigue at 2-yr	97 (32.7%)	30 (4.7%)	<0.0001

Continuous data are expressed as means ± SD, whereas categorical data are presented as frequency counts (percentages).

BMI, body mass index; FIB-4, Fibrosis-4; MASLD, metabolic dysfunction-associated steatotic liver disease.

a Defined as FIB-4 ≥2.67 or liver stiffness ≥12 kPa.

b Defined as fatigue or asthenia mentioned in medical history.

**Table 4.** Patient-reported outcomes of MASLD patients stratified by the presence of significant fatigue

Score (range)	Significant fatigue	No significant fatigue	*P*
CLDQ-NASH			
Abdominal symptoms (1–7)	3.99 ± 1.51	5.43 ± 1.38	<0.0001
Activity/energy (1–7)	3.92 ± 1.18	5.62 ± 1.10	<0.0001
Emotional health (1–7)	3.58 ± 1.16	5.28 ± 1.09	<0.0001
Fatigue (1–7)	2.82 ± 1.05	4.90 ± 1.19	<0.0001
Systemic symptoms (1–7)	3.53 ± 1.16	5.17 ± 1.13	<0.0001
Worry (1–7)	4.34 ± 1.48	5.68 ± 1.23	<0.0001
Total (1–7)	3.70 ± 0.88	5.35 ± 0.87	<0.0001
FACIT-F			
Physical well-being (0–28)	15.6 ± 5.4	24.3 ± 3.4	<0.0001
Emotional well-being (0–24)	12.1 ± 4.7	17.7 ± 4.2	<0.0001
Social well-being (0–28)	18.9 ± 6.0	20.8 ± 6.1	<0.0001
Functional well-being (0–28)	16.0 ± 5.1	21.3 ± 5.0	<0.0001
Fatigue scale (0–52)	19.3 ± 6.6	41.9 ± 6.4	<0.0001
Total (0–160)	81.9 ± 18.5	126.1 ± 17.0	<0.0001
WPAI:SHP			
Work productivity impairment (1-0)	0.411 ± 0.349	0.120 ± 0.224	<0.0001
Absenteeism (1-0)	0.048 ± 0.143	0.026 ± 0.119	0.0017
Presenteeism (1-0)	0.365 ± 0.328	0.096 ± 0.190	<0.0001
Activity impairment (1-0)	0.396 ± 0.328	0.117 ± 0.207	<0.0001

Data are expressed as means ± SD, whereas categorical data are presented as frequency counts (percentages).

CLDQ-NASH, Chronic Liver Disease Questionnaire-NASH; FACIT-F, Functional Assessment of Chronic Illness Therapy-Fatigue; MASLD, metabolic dysfunction-associated steatotic liver disease; WPAI:SHP, Work Productivity and Activity Impairment, Specific Health Problem.

### Longitudinal findings on significant fatigue

Among patients with 1-year follow-up data, a significant association was observed between baseline and follow-up fatigue status. Specifically, 39% of patients who had significant fatigue at baseline continued to experience significant fatigue at 1-year follow-up, compared with 6% who developed de novo significant fatigue (*P* < 0.0001; Table 3). This pattern was similar in the subsample with a 2-year follow-up, where the rates of persistent vs de novo fatigue were significantly different (33% vs 5%, respectively, *P* < 0.0001; Table 3). When examining patient groups defined by the presence of significant fatigue at baseline and 1-year follow-up, 4 distinct categories emerged: 63% never experienced significant fatigue, 4% developed de novo significant fatigue, 20% had their baseline significant fatigue resolve, and 13% had persistent fatigue. Notably, PRO scores were the lowest among patients with persistent fatigue, followed by those who developed de novo significant fatigue (all *P* < 0.0001; Figure 1b). Furthermore, mirroring the trends observed with pruritus, patients who never experienced significant fatigue had the highest PRO scores at 1-year follow-up. The observed values were significantly higher compared with those of patients whose baseline significant fatigue had resolved, with impairments of up to 14% across all nonfatigue PRO scores (*P* < 0.05; Figure 1b).

### Multivariable analysis

In multivariable analysis, several factors were independently associated with an increased risk of both baseline significant pruritus and fatigue. These variables included female sex, type 2 diabetes, depression, abdominal pain, lack of regular exercise, and the presence of significant fatigue or pruritus, respectively (all *P* < 0.05; Table 5). In addition, significant fatigue was also linked to younger age, with an OR of 0.97 per year (95% CI: 0.96−0.98; *P* < 0.0001; Table 5). Notably, there was no independent association between significant pruritus or fatigue and obesity or advanced fibrosis (Table 5). Independent predictors of a higher likelihood of resolution of significant pruritus included the absence of significant fatigue at baseline and the absence of abdominal pain, with ORs of 0.36 (95% CI: 0.22−0.59) and 0.60 (95% CI: 0.39−0.91), respectively (both *P* < 0.05; Table 5). Conversely, independent predictors of resolution of significant fatigue were the absence of significant pruritus at baseline and the absence of depression, with ORs of 0.40 (95% CI: 0.26−0.62) and 0.50 (95% CI: 0.32−0.79), respectively (both *P* < 0.01; Table 5). Interestingly, the same factors, when reversed, were independently associated with an increased risk of developing de novo significant pruritus or significant fatigue (Table 5). Furthermore, the presence of type 2 diabetes was an independent risk factor for de novo significant pruritus, with an OR of 1.66 (95% CI: 1.12–2.44, *P* = 0.01; Table 5).

**Table 5.** Independent predictors of significant pruritus and fatigue in MASLD patients

Predictor/outcome	Significant pruritus		Significant fatigue	
Significant <pruritus/fatigue> at baseline	Odds ratio (95% CI)	*P*	Odds ratio (95% CI)	*P*
Age, per yr	1.004 (0.994–1.013)	0.43	0.973 (0.963–0.983)	<0.0001
Male sex (reference: female)	0.79 (0.64–0.97)	0.0243	0.52 (0.41–0.65)	<0.0001
Advanced fibrosis	1.10 (0.85–1.43)	0.48	1.14 (0.86–1.51)	0.38
Type 2 diabetes	1.29 (1.04–1.60)	0.0215	1.35 (1.07–1.71)	0.0118
Obesity	1.13 (0.89–1.42)	0.31	1.21 (0.94–1.56)	0.14
Depression	1.45 (1.12–1.88)	0.0048	2.76 (2.11–3.61)	<0.0001
Significant <fatigue/pruritus>	2.38 (1.91–2.96)	<0.0001	2.39 (1.92–2.97)	<0.0001
Abdominal pain	1.79 (1.42–2.26)	<0.0001	2.82 (2.21–3.58)	<0.0001
Regular exercise	0.80 (0.65–0.99)	0.0356	0.70 (0.56–0.87)	0.0016

Resolution of <pruritus/fatigue> at 1-yr	Odds ratio (95% CI)	*P*	Odds ratio (95% CI)	*P*
Age, per yr	1.003 (0.983–1.023)	0.76	0.994 (0.974–1.015)	0.59
Male sex (reference: female)	1.10 (0.72–1.68)	0.67	1.31 (0.83–2.08)	0.25
Advanced fibrosis	0.79 (0.48–1.30)	0.36	1.11 (0.64–1.94)	0.71
Type 2 diabetes	0.80 (0.52–1.24)	0.31	1.54 (0.97–2.46)	0.07
Obesity	1.07 (0.67–1.72)	0.77	0.60 (0.35–1.01)	0.05
Depression	1.03 (0.64–1.66)	0.91	0.50 (0.32–0.79)	0.0032
Significant < fatigue/pruritus >	0.36 (0.22–0.59)	<0.0001	0.40 (0.26–0.62)	<0.0001
Abdominal pain	0.60 (0.39–0.91)	0.0158	0.76 (0.49–1.17)	0.21
Regular exercise	1.36 (0.90–2.05)	0.14	0.90 (0.57–1.42)	0.65

De novo <pruritus/fatigue> at 1-yr	Odds ratio (95% CI)	*P*	Odds ratio (95% CI)	*P*
Age, per yr	1.019 (1.000–1.037)	0.05	0.982 (0.953–1.011)	0.23
Male sex (reference: female)	0.8 (0.54–1.18)	0.25	0.62 (0.33–1.14)	0.12
Advanced fibrosis	0.88 (0.55–1.42)	0.60	1.60 (0.81–3.15)	0.18
Type 2 diabetes	1.66 (1.12–2.44)	0.0111	1.04 (0.55–1.94)	0.91
Obesity	0.86 (0.57–1.28)	0.45	1.52 (0.75–3.07)	0.25
Depression	0.99 (0.59–1.67)	0.97	2.51 (1.22–5.15)	0.0121
Significant < fatigue/pruritus >	2.55 (1.55–4.18)	0.0002	2.25 (1.21–4.19)	0.0102
Abdominal pain	1.99 (1.29–3.05)	0.0017	1.40 (0.70–2.80)	0.34
Regular exercise	0.86 (0.59–1.25)	0.42	0.80 (0.44–1.47)	0.48

MASLD, metabolic dysfunction-associated steatotic liver disease; CI, confidence interval.

## DISCUSSION

This registry-based study reveals a notable prevalence of pruritus and fatigue in MASLD, affecting 37% and 33% of Turkish MASLD patients enrolled in this registry. These symptoms frequently co-occurred and were strongly interrelated. Importantly, patients experiencing these symptoms reported significantly worse PROs across multiple domains compared with those without these symptoms. Over a follow-up period of 1–2 years, pruritus and fatigue often persisted, continuing to be associated with impaired PROs. These findings address key research questions concerning the prevalence, impact, and longitudinal course of pruritus and fatigue in MASLD. The symptoms affect a substantial proportion of patients, exerting a profound negative impact on their well-being and functioning. In addition, the frequent persistence of these symptoms and the associated impairments underscore their chronic nature.

The pathophysiology of pruritus and fatigue in MASLD is not entirely elucidated. Mechanisms contributing to hepatogenic pruritus, extensively investigated in primary biliary cholangitis, encompass heightened opioidergic tone, bile acid accumulation, hyperferritinemia, and elevated autotaxin and lysophosphatidic acid levels (22,23). Whether these pathways are also implicated in MASLD warrants further exploration. Notably, Xu et al (24) recently observed elevated baseline interleukin (IL)-31 levels in both NASH and primary biliary cholangitis, with more pronounced increases in the latter, and found IL-31 levels correlated with pruritus severity. In NASH patients receiving the farnesoid X receptor agonist cilofexor, IL-31 levels increased dose-dependently, especially in those experiencing more severe pruritus, which has implications for pruritus management. The authors proposed that increased IL-31 mRNA expression in hepatocytes from patients with NASH suggests hepatocytes may contribute to elevated IL-31 levels (24). Further research is necessary to confirm the role of IL-31 as a potential biomarker for pruritus in MASLD. Regarding fatigue, the liver plays a crucial role in its pathogenesis due to its unique regulation of production, storage, and release of energy substrates (25). In addition, the liver engages in cross-talk with key organs responsible for fatigue, including skeletal muscle and the brain (25,26). The highly significant association between pruritus and fatigue observed in our study suggests potential shared underlying mechanisms. In this context, individuals with chronic pruritus frequently encounter sleep disturbances, which can directly result in fatigue (27). Systemic inflammation may also play a role in both symptoms (27), and individuals experiencing fatigue are at a significantly heightened risk of compromised metabolic health and overall mortality (28).

Although no patients received treatment specifically for pruritus or fatigue, there was a noted improvement in some patients but not in others. This is not surprising as our current understanding is that fatigue and pruritus associated with liver disease many times only respond to treatment of the underlying liver disease and does not always correlate with the severity of liver disease (8). Such that in this study, we are most likely observing the natural history of MASLD and MASH where approximately 15%–33% may experience regression of their liver disease, while 19%–60% may experience disease progression (29,30). These highly variable outcomes are mostly dependent on a person's environment, disease stage, and weight loss/gain. In this context, assessing fatigue and pruritus may be helpful in assessing disease status, although this suggestion will need further investigation.

Nonetheless, it is important to prioritize the assessment of pruritus and fatigue in patients with MASLD and to acknowledge their significant impact on PROs. As noted above, the cornerstone of managing these symptoms involves treating the liver disease itself through lifestyle modifications, such as promoting regular physical activity, achieving a healthy weight, and optimizing glycemic control in patients with type 2 diabetes. Given that patients with MASLD and significant pruritus or fatigue exhibited a higher prevalence of comorbidities including anxiety, depression, hypertension, hyperlipidemia, and sleep apnea, a comprehensive management strategy should incorporate mental health support, cardiometabolic condition management, addressing sleep disturbances, cognitive-behavioral therapy, and providing psychosocial support (31,32). Symptom-specific interventions for pruritus with a trial use of pharmacological or topical therapies may be warranted (33,34), although specific data in the context of MASLD are currently limited. Ultimately, managing pruritus and fatigue in MASLD requires a holistic, personalized approach and close collaboration among healthcare professionals to enhance PROs. Further research is essential to establish evidence-based guidelines for managing these symptoms specifically in the context of MASLD.

The strengths of this study include a large, well-characterized registry-based sample, a prospective design with a 1–2 years follow-up period, and comprehensive multidimensional PRO assessments. However, a significant limitation is that we did not collect data on potential underlying mechanisms of pruritus and fatigue. In addition, it is important to note that since the sample was drawn from referral centers, the prevalence of symptoms may be higher than in community-based MASLD populations. The instruments used to define the symptoms are generally subject to the recall bias, while the chosen cutoff values, albeit consistent with some prior studies, lacked independent validation. The clinical data collected for the registry were limited and, in particular, lacked potentially relevant confounding factors, such as a history of skin disorders or medication use, making their impact on symptom resolution, and/or changes in PRO scores unclear.

In conclusion, this study underscores the high prevalence of pruritus and fatigue and their detrimental impact on PROs within a cohort of Turkish patients with MASLD from the GLR. Further research is essential to elucidate the underlying mechanisms and develop optimal management strategies for these symptoms. Meanwhile, hepatologists should prioritize the identification and management of pruritus and fatigue to improve MASLD patients' well-being and functional status.

## CONFLICTS OF INTEREST

**Guarantor of the article:** Zobair M. Younossi, MD.

**Specific author contributions:** Y.Y.: study design, data interpretation, manuscript writing and critical editing. C.K.: data interpretation, manuscript writing and critical editing. A.R.: database administration and manuscript writing and critical editing. M.S.: study design, statistical analysis, data interpretation, manuscript writing and critical editing. Z.Y.: study design, data interpretation, manuscript writing and critical editing.

**Financial support:** The study was partially supported by the Global NASH/MASH Council and Beatty Liver and Obesity Fund.

**Potential competing interests:** Z.Y. has received research funding and/or serve as consultant to Intercept, CymaBay, Boehringer Ingelheim, BMS, GSK, NovoNordisk, Ipsen, AstraZeneca, Siemens, Madridgal, Merck, and Abbott. All other authors declare that they have no conflict of interest.

**Data availability statement:** The data that support the findings of this study are available on request from the corresponding author. The data are not publicly available due to privacy or ethical restrictions.

**Patient consent statement:** You are being asked to be in a research study whose purposes are to collect clinical and patient-reported outcome (PRO) information for patients with chronic liver disease before and/or during treatment. The data we collect from the questionnaires will also help researchers assess your experience with chronic liver disease and its treatment (if applicable). Your information will be completely confidential and will only be seen by researchers at your institution. We will take all precautions so that the information we collect from you is kept private and used only for the research study we are discussing.

**Study Highlights**

**WHAT IS KNOWN**

✓ Although metabolic dysfunction-associated steatotic liver disease is associated with a decreased health-related quality of life (HRQL), the impact of pruritus and fatigue on HRQL is a newer area of study.

**WHAT IS NEW HERE**

✓ We found that 40% of patients from Turkey who had metabolic dysfunction-associated steatotic liver disease reported having pruritus, while 30% reported having fatigue whose presence had a significant negative impact on HRQL. Female sex, type 2 diabetes, depression, abdominal pain, and lack of regular exercise were more often associated with the presence of pruritus and fatigue.
✓ Regular assessment and management are essential to improve patients' well-being and functioning.
